# Comparison of Culture and Molecular Identification of Bacteria in Chronic Wounds

**DOI:** 10.3390/ijms13032535

**Published:** 2012-02-23

**Authors:** Daniel D. Rhoads, Randall D. Wolcott, Yan Sun, Scot E. Dowd

**Affiliations:** 1 Southwest Regional Wound Care Center, Lubbock, TX 79410, USA; 2 Texas Tech University Health Sciences Center School of Medicine, Lubbock, TX 79415, USA; 3 Research and Testing Laboratory, Lubbock, TX 79407, USA; 4 Pathogenius Laboratory, Lubbock, TX 79407, USA

**Keywords:** wound, diagnosis, biofilm, molecular

## Abstract

Clinical diagnostics of chronic polymicrobial infections, such as those found in chronic wounds, represent a diagnostic challenge for both culture and molecular methods. In the current retrospective study, the results of aerobic bacterial cultures and culture-free bacterial identification using DNA analyses were compared. A total of 168 chronic wounds were studied. The majority of bacteria identified with culture testing were also identified with molecular testing, but the majority of bacteria identified with the molecular testing were not identified with culture testing. Seventeen (17) different bacterial taxa were identified with culture, and 338 different bacterial taxa were identified with molecular testing. This study demonstrates the increased sensitivity that molecular microbial identification can have over culture methodologies, and previous studies suggest that molecular bacterial identification can improve the clinical outcomes of patients with chronic wounds.

## 1. Introduction

### 1.1. Chronic Wounds

Chronic wounds, wounds that fail to progress through normal wound healing trajectory, are a large and growing problem for an already burdened healthcare system. The major chronic wounds (diabetic foot ulcers, venous leg ulcers, pressure sores, nonhealing surgical wounds) are all increasing at alarming rates due to sedentary lifestyles, obesity and, most importantly, double digit increases in diabetes each year. The total cost of chronic wounds is difficult to measure but has been estimated in over 25 billion dollars a year, but even more importantly is the patient impact resulting in loss of limb and eventually loss of life for far too many patients [1,2].

Wound care providers treat wounds as if the bioburden is one of the major barriers to wound healing. Debridement, antibiotics, and topical antiseptics are routinely used in managing the microbes that are universally found on chronic wounds. Yet, the only microbial diagnostic tool available to wound care providers until recently has been the routine clinical culture. The role of bacteria in chronic wounds and the appropriateness of using antimicrobial agents in the routine management of chronic wounds is a source of continual debate in the wound care community [3–5]. It is reasonable to consider that improving the methods of microbial detection and identification may be more successful in helping the clinician deal with this important healthcare problem.

### 1.2. Bacterial Biofilms

It is now recognized that the overwhelming majority of bacteria in their natural habit exist in a biofilm phenotype [6]. Chronic biofilm infections affect every organ system, including the integumentary system [7–9]. Bacterial biofilms in chronic wounds affect millions in the United States each year [1].

Biofilms are inherently different from the bacterial clones that are cultivated in laboratories. In order to standardize testing of bacteria, the convention has been to examine free-floating clonal populations of a single species during its logarithmic growth phase. Unfortunately, biofilms are archetypically surface-associated groups, often comprised of multiple genera or kingdoms of organisms, held together by an extracellular matrix; and the growth conditions and population within the biofilm can be spatially and temporally variable [10]. By definition, biofilms are more resistant to antimicrobial agents than the planktonic cells cultivated in the laboratory, and the level of resistance can be two or three orders of magnitude greater for cells within a biofilm [11]. Biofilms are also resistant to host immunity defenses [8]. Unfortunately, the patients who develop chronic wounds are often immune compromised in some way (e.g., poor circulation or hyperglycemia), which facilitates the establishment of bacterial biofilm communities and makes elimination of the biofilm all the more difficult.

Multiple bacteria genera comprise the biofilm in chronic wounds [9,12,13], and some of these bacterial taxa seem to commonly co-occur with each other [12,14]. In oral biofilm disease, co-occurrence of bacterial taxa has been well described spatially and temporally, and its relevance to the biofilm’s pathogenesis has been described [10]. This description has not yet been established in chronic wounds, but it has caused some to question the traditional paradigm of infection. The lack of a single identifiable bacterial species causing an infection in chronic wounds has caused some to question the relevance of Koch’s postulates as it pertains to chronic wound infections [15]. It has been proposed that the cause of chronic wound infections may not be able to be reduced to a single species of bacteria, but perhaps the collective polymicrobial biofilm community may be required for effective chronic pathogenesis by the bacteria [15–17].

### 1.3. Bacteria in Wounds

The debate over which organisms are harmless, helpful, or hateful to humans is continually being investigated and debated. A prerequisite assumption to this debate is that the presence of bacteria in or on humans is accurately being detected and identified. Traditionally, the presence of bacteria in or on humans during health or disease has been determined by growing the bacteria in culture. Indeed, Koch’s postulates and infectious disease dogma are anchored in culture technology. However, molecular biology has proven that culture methodologies can be markedly less sensitive to bacterial detection than DNA detection methodologies [13]. Also, the identification of bacteria is shifting away from the traditional metabolic biochemical testing of cultures and moving towards genetic or proteomic identification of the bacteria [13,18,19]. The advent of molecular microbiology has caused medicine to reexamine Koch’s postulates [20,21]. Also, science’s growing understanding of biofilms’ role in disease, has led others to challenge the relevance of Koch’s postulates in biofilm wound infections [15]. Some have recognized that infections can be caused by bacteria that are not especially virulent but which commonly comprise part of the benign or symbiotic human microbiome. However, these normally benign organisms are capable of filling the niche provided to them by an impaired host, thereby causing disease and being implicated as a pathogen [20]. Others suggest that it may not be a single species that causes chronic wound infections, but the collective biofilm can assert a pathogenicity that the component parts are less capable of exerting [15–17].

### 1.4. Molecular Identification of Bacteria

Molecular technologies have enabled investigators to examine human microbiota more deeply and sensitively than culture testing has allowed [16,17]. In our experience, these molecular tests can reveal an order of magnitude greater number of bacterial species in chronic wounds than culture results demonstrate. We have used molecular microbial diagnostic information to develop personalized medicine methods in wound care, which has improved patient outcomes [22,23].

It is encouraging that these new molecular microbial diagnostic tests are enabling improvement in patient care, but it is also necessary to continue to formally compare these newer methods with traditional culture methods. Examining culture and molecular results in parallel is necessary in order to compare and contrast the methods’ abilities to detect bacteria in humans and to continue to evaluate the clinical relevance of molecular testing’s increased sensitivity. This study is an early attempt to compare clinical laboratory results of parallel testing of aerobic cultures and culture-free bacterial identification using 16S rDNA sequencing.

## 2. Study Design and Methods

This study was performed retrospectively on subjects who had undergone parallel bacterial analysis of wound samples. Initially, the results obtained by the Bacterial Identification Methods (2.3) were used clinically to guide patient care.

### 2.1. Inclusion Criteria

Subjects were included in the study if they met the following criteria:

1. The presence of a chronic wound (“Chronic wound” is defined as a wound that fails to progress through the normal healing trajectory);
2. Debridement required as part of standard of care wound management;
3. The medical record revealed that parallel samples from the wound were sent for both, culture and molecular testing.

### 2.2. Sample Collection

Samples were collected from 168 subjects’ chronic wounds at the Southwest Regional Wound Care Center (Lubbock, Texas, USA) in accordance with Western Institutional Review Board protocol number 20062347.

### 2.3. Bacterial Identification Methods

#### 2.3.1. Culture Identification of Bacteria

Samples were tested using routine aerobic culture techniques in a non-hospital CLIA certified laboratory in Lubbock, Texas, USA.

#### 2.3.2. Molecular Identification of Bacteria using 16S Sequencing

Samples were prepared and analyzed by PathoGenius Laboratory (Lubbock, Texas, USA), a College of American Pathologists accredited laboratory.

##### 2.3.2.1. DNA Extraction

The debridement samples were centrifuged and then suspended in 500 μL RLT buffer (Qiagen, Valencia, CA) (with b-mercaptoethanol). A sterile 5 mm steel bead (Qiagen, Valencia, CA) and 500 μL sterile 0.1 mm glass beads (Scientific Industries, Inc., NY, USA) were added for complete bacterial lysis in a Qiagen TissueLyser (Qiagen, Valencia, CA), run at 30 Hz for 5 min. Samples were centrifuged briefly and 100 μL of 100% ethanol added to a 100 μL aliquot of the sample supernatant. This mixture was added to a DNA spin column, and DNA recovery protocols were followed as instructed in the QIAamp DNA Mini Kit (Qiagen, Valencia, CA) starting at step 5 of the Tissue Protocol. DNA was eluted from the column with 30 μL water and samples were diluted accordingly to a final concentration of 20 ng/μL.

##### 2.3.2.2. Partial Ribosomal Amplification

The modified 16S Eubacterial primers 28F, 5′-GAG TTT GAT CNT GGC TCA G-3′ and 519R, 5′-GTN TTA CNG CGG CKG CTG-3′ were used for amplifying the 500 bp region of 16S rRNA genes. The primer sets used for FLX-Titanium amplicon pyrosequencing were designed with adding linker A and 8 base pair subject-specific barcode sequence at the 5′ end of forward primers as follow: 28F-A, 5′-CCA TCT CAT CCC TGC GTG TCT CCG ACT CAG-barcode-GAG TTT GAT CNT GGC TCA G-3′. The biotin and linker B sequence at the 5′ end of reverse primer 519R-B: 5′-Biotin-CCT ATC CCC TGT GTG CCT TGG CAG TCT CAG GTN TTA CNG CGG CKG CTG-3′. HotStarTaq Plus Master Mix Kit (QIAGEN, CA, USA) was used for PCR under the following conditions: 95 °C for 5 min followed by 35 cycles of 95 °C for 30 s; 54 °C for 40 s and 72 °C for 1 min, a final elongation step at 72 °C for 10 min was also included. The PCR products were cleaned by using Diffinity Rapid Tip (Diffinity Genomics, Inc, West Henrietta, NY) and pooled. The small fragments were removed by Agencourt Ampure Beads (Beckman Coulter, CA, USA).

##### 2.3.2.3. Massively Parallel bTEFAP Titanium

Bacterial tag-encoded FLX-Titanium amplicon pyrosequencing (bTEFAP) was performed as described previously [17]. In preparation for FLX-Titanium sequencing (Roche, Nutley, New Jersey), a sample of double-stranded DNA was combined with DNA capture beads, and amplified by emulsion PCR. After bead recovery and bead enrichment, the bead attached DNAs were denatured with NaOH, and sequencing primers (Roche) were annealed. A 454 sequencing run was performed on a GS PicoTiterPlate (PTP) using the Genome Sequencer FLX System (Roche). All FLX procedures were performed using Genome Sequencer FLX System manufacturer’s instructions (Roche).

#### 2.3.3. Polymerase Chain Reaction

The diagnostic panel for quantitative real-time PCR consists of Pseudomonas aeruginosa, Group B Streptococcus agalactiae (Group B Streptococcus), Streptococcus pyogenes (Group A Streptococcus), Staphylococcus aureus, Klebsiella pneumonia, Serratia marcescens, Candida albicans, Enterococcus faecalis, Enterococcus faecium. The enterococci were reported collectively in this paper as “Group D Enterococcus”. The quantitative real-time PCR was performed by a Roche Light Cycler 480 as described previously [24]. The PCR reactions were as follows: deactivation, 95 °C for 10 s; amplification, 35 cycles of 95 °C for 15 s, 60 °C for 1 min.

### 2.4. Description of Analysis

The concordance or discordance of molecular results with culture results were identified as “concordant”, “discordant”, or “partially concordant”. “Concordance” was defined as detecting the same bacterium/bacteria using molecular testing as were detected using culture testing. “Discordance” was defined as not detecting the bacterium/bacteria using molecular testing when the bacterium/bacteria was identified using culture testing. “Partial concordance” was possible when multiple bacteria were identified by culture. In partially concordant subjects’ samples, one or more of the bacteria identified by culture were also identified by molecular testing and one or more of the bacteria identified by culture were not identified by molecular testing.

## 3. Results of Bacterial Identification

One hundred sixty-eight (168) wounds from 168 subjects were analyzed by both culture and molecular testing including 24 decubitus ulcers, 40 diabetic extremity wounds, 23 non-healing surgical sites, 49 venous leg ulcers, and 32 wounds from trauma or an abscess. The most common results using both culture and molecular testing are reported by wound type in Table 1. Overall, when considering all 168 wounds, the most common organism identified using both culture and molecular testing was *Staphylococcus aureus*. In total, 17 different bacterial taxa were identified by culture. These organisms, the frequency of culture of each organism, and the corresponding frequency of detection by molecular testing are reported in Table 2. When considering all 168 wounds, 338 different bacterial taxa were identified by molecular testing. Of the 338 bacterial taxa, the 20 most frequently identified taxa and the frequency of their detection are reported in Table 3. The majority of the taxa were detected to be less than 1% of the total microbiota in any given wound. Briefly, culture commonly identified *Staphylococcus*, *Enterococcus*, *Serratia*, and *Pseudomonas*. Molecular testing commonly identified *Staphylococcus*, *Finegoldia*, *Corynebacterium*, *Anaerococcus*, *Bacteroides*, and *Serratia*.

A subset of results are reported more exhaustively in Table 4. The table compares the results of 10 samples as determined by culture and 16S sequencing. Additionally, the results from PCR assays, 18S sequencing detection of yeast, and incidental identification of yeast during bacterial culture are included in the table. Different taxa were determined to be major contributors to the microbiota in different wounds. Some sample results were very similar across testing methods, and other samples revealed a large amount of variability from method to method. Overall, the table demonstrates that deep 16S/18S sequencing detects organisms that are detected using other molecular methods (PCR) and culturing methods.

From the culture-based diagnostics, a total of 11 (7%) chronic wounds were found to have 3 different bacterial taxa, 40 of the samples (24%) had 2 bacterial taxa, and 95 of the wounds (57%) had a single bacterium cultured. 22 of the wounds (13%) were found to have no culturable bacteria. Seventeen (17) different bacterial taxa were identified using culture based methods (Table 2).

Molecular testing identified 338 different bacterial taxa (mean of 6.9 different bacterial taxa per wound with a range of 1–33) in the 168 wounds. None of the chronic wounds were reported as negative based upon molecular diagnostics. It is of note that out of the top 20 most ubiquitous bacteria identified with molecular methods, 9 of these taxa are anaerobes (Table 3). Anaerobic cultures were not performed.

Of the 168 paired samples, 131 of the samples (78%) demonstrated some level of concordance between culture and molecular results. In 105 of the samples (63%), the molecular results were concordant with the culture results. Twenty-six (26) samples (15%) had partial concordance. Fifteen (15) samples (9%) were discordant. Twenty-two (22) samples (13%) were negative by culture testing but positive with molecular testing. In these 22 samples, molecular testing revealed these bacteria: Bacteroidetes (unknown genus), *Bacteroides*, *Finegoldia*, *Peptoniphilus*, *Anaerococcus*, *Propionibacterium*, *Ruminococcus*, *Prevotella*, *Eubacterium*, *Streptococcus*, *Corynebacterium*, *Arthrobacter*, *Acinetobacter*, *Paracoccus*, and *Micrococcus*. Many of these genera are obligate anaerobes.

Of the 105 samples with complete concordance, 55 samples were in agreement between culture and molecular testing as to which bacteria were most prevalent in the sample. In 15 of the 105 samples, culture and molecular testing were not in agreement as to which bacteria were most prevalent in the sample, but the cultured organism(s) was determined to be one of the 3 most prevalent organisms by using molecular testing. In the other 35 samples, the cultured bacteria were identified as representing a minor population (not one of the 3 most prevalent bacteria within the sample) when using molecular methods.

Of the 41 samples with partial concordance or complete discordance, the bacteria that were cultured but not detected by molecular testing are were as follows: *Enterococcus* spp. (19 times), *Staphylococcus* spp. (6 times), *Pseudomonas* spp. (4 times), *Serratia marcescens* (3 times), *Enterobacter* (3 times), *Citrobacter* (3 times), *Proteus* (1 time), *Xanthamonas* (1 time), and *Klebsiella* (1 time). Thirty-two (32) of these 41 samples were determined by molecular methods to have strong anaerobic contribution to the microbiota (primarily *Bacteroides*, *Peptoniphilus*, *Finegoldia*, *Anaerococcus*, and *Clostridium*).

More exhaustive results of a subset of 10 samples are reported in Table 4 to demonstrate the clinical relevance of the two methods. The culture results and 16S sequencing results are reported. In addition to these results, incidental findings of yeast by culture, results from PCR assays for select organisms, and results of 18S sequencing for yeast [25] are reported. Not all of the organisms detected using 16S/18S sequencing are reported. Results are reported for those organisms that met at least one of the following criteria: (1) contributed to 10% or more of the microbiota in the sample; (2) were attempted to be detected using PCR or (3) were identified by culture. The lower sensitivity of bacterial culturing is demonstrated in this table. A PCR-targeted species was detected in six samples by both PCR and 16S sequencing (Samples 2, 3, 4, 7, 9, 10) but was not detected by culture. Cultures were able to detect bacteria for which PCR detection was not attempted (Sample 5 & 7). This demonstrates the broad sensitivity of cultures when compared to PCR testing. PCR testing is highly sensitive but requires the laboratory to specifically test for each bacterium of interest with an individualized assay. 16S/18S sequencing has both broad sensitivity (similar to culturing) and high sensitivity (similar to PCR). Molecular methods (PCR and 16S/18S sequencing) are sensitive and specific. PCR’s specificity is demonstrated by the results of Sample 4. Sample 4 was negative for *P. aeruginosa*, but another pseudomonad species was detected by both culture and 16S sequencing. The only discrepancy between 16S sequencing and PCR is in Sample 1, in which *S. marcescens* and Group D *Enterococcus* were detected by 16S sequencing and not detected by PCR. This contradiction in results is likely due to the high sensitivity of deep 16S sequencing and not due to poor specificity of 16S sequencing. Congruent with that explanation, 16S sequencing detected that *S. marcescens* and Group D *Enterococcus* comprised a very small portion of the total microbiota in Sample 1. Overall, the table demonstrates that deep 16S/18S sequencing detects organisms that are detected using other molecular methods (PCR) and culturing methods.

## 4. Analysis and Discussion of Results

Culture results and 16S DNA sequencing results were compared more exhaustively in a subset of samples (Table 4). Quantitative PCR results were also reported, which help to clarify discrepancies in the results between culturing and 16S sequencing. PCR also helped to verify the results of cultures and 16S sequencing. The results of the additional PCR testing demonstrate close correlation between the molecular results: PCR and 16S sequencing. Culture results appear to be less sensitive than the molecular testing methods, as has been well established. PCR testing results are similar to the 16S sequencing results. However, 16S sequencing has the potential to detect almost any clinically relevant bacterium, even bacteria that are not yet well described. For example, Samples 2, 8, and 9 were determined to have a significant portion of unknown bacteria in the wound microbiota. In contrast, PCR assays need to be designed to investigate each bacterium of interest. The rapidity of PCR testing is a potential advantage over both culturing and 16S sequencing. However, the advent of MALDI-TOF identification of bacterial cultures is likely to significantly decrease the turn around time for culture results. Still, cultures will continue to take greater than 24 h, but some molecular tests can potentially be complete within one working day. The turn around time for molecular testing is likely to continue to decrease as molecular technologies and computational hardware improve. Molecular methods maintain an advantage, at least theoretically, in turn around time; and molecular methods are more sensitive than cultures.

In the present study, the microbes that were both commonly predominant in wounds according to molecular testing and also not identified using culture methods were often bacteria that were obligate anaerobes. The results support the interpretation that aerobic cultures alone (as in this study) fail to resolve the complexity of the polymicrobial nature of chronic wounds. Although anaerobes were not attempted to be cultured, it is reasonable to estimate that the frequency with which anaerobes would have been detected would have been similar to the frequency with which aerobes were detected. As reported previously, although anaerobes are difficult to culture, they often comprise a large portion of the microbiota of chronic wounds [13,16]. The detection of aerobes by culture was largely in agreement with the molecular results, but molecular testing often found many more bacteria and bacteria that were larger contributors to the microbiota than those detected by culture. Molecular diagnostics are able to resolve a more accurate and actionable diagnosis [22,23,26]. This study contributes to a large body of evidence that recognizes the existence of anaerobes as an important population in chronic wound biofilms [13,16,27,28]. Evidence is also abundant in the scientific literature that chronic wounds are highly polymicrobial infections, which supports the findings made with the molecular methods used in this study [12,23,29,30].

Bowler and Davies conducted one of the most comprehensive culture-based surveys of chronic wounds with methods specifically designed to enrich for and detect anaerobes, which revealed that anaerobes were highly prevalent [16,31]. In this study, the 41 samples showing discordance or partial concordance between culture and molecular testing commonly had large anaerobic bacterial populations as determined using molecular testing. The 16S DNA from these anaerobes may have diluted the concentration of 16S DNA from the aerobes in the sample that went undetected. Aerobic organisms that were not detected by molecular testing may have been present below the level of sensitivity. If this is true, then these bacteria should be able to be detected in the future by sequencing a greater number of 16S rDNA molecules from the samples, thereby increasing the test’s sensitivity. Another potential cause of decreased sensitivity using 16S rDNA interrogation is the potentially large portion of the extracted DNA that is human and not bacterial in origin [13]. It is also important to note, if anaerobes are inadequately investigated within a wound (such as with aerobic-only cultures as in this study), clinicians could incorrectly infer that minor contributors to the bacterial population (cultured aerobic bacteria) comprise a substantial portion of the wound microbiota (e.g., Table 4, Samples 2 and 6). This could result in targeting the “wrong” bacteria for antimicrobial therapy. It is clinically important to investigate the presence of anaerobes in chronic wounds.

Molecular testing identified each taxon more frequently than culturing with the exception of *Enterococcus* and *Escherichia coli* (Table 2). Enterococci were cultured from 35 samples but only 28 samples were positive using molecular testing. We have found similar results in another study (pending publication) and can identify several possible reasons for this finding: potentially higher sensitivity of the culture method beyond the sensitivity of the molecular method, misidentification of the bacterium by culture or by molecular testing, or contamination of the sample.

There were 22 culture-negative/molecular-positive samples and no culture-positive/molecular-negative samples. These findings could be a result of several differences in the testing methodologies. Either culturing produces false negative results (poor sensitivity) or molecular testing produces false positive results (poor specificity). Or, some combination of the two proposed explanations is possible. The verification of 16S sequencing results by PCR testing (Table 4) helps to rule out the cause of the discrepancies being linked to false positive results using 16S analysis. The sensitivity of culturing can be lowered by the presence of viable but not culturable (VBNC) bacteria, including the VBNC persister bacteria within biofilms [32]. While the potential bias exists for the detection of nonviable bacteria with molecular methods, it has been reported that nucleic acid artifact from dead bacteria do not reside for long periods of time in an active host environment [33,34]. Published studies suggest that using molecular bacterial detection results has the potential to improve clinical outcomes of patients with chronic infections more than the use of culture results [22,23,26].

A potential limitation of molecular diagnostics is the inability to test for phenotypic antibiotic sensitivity. It is important to consider the method in which phenotypic antibiotic testing is performed. Culture sensitivities are performed using planktonic phenotype bacteria and not biofilm phenotype bacteria. Obtaining the phenotypic sensitivity of biofilms is much more cumbersome than traditional susceptibility testing [35–37]. Biofilm bacteria are present in chronic wounds, and the resistance of this biofilm phenotype to antimicrobial agents can be orders of magnitude greater than the traditional laboratory culture methods can demonstrate [38–40]. Although not reported in this study, molecular methods are used in clinical laboratories to detect notable antibiotic resistance genes such as methicillin resistance and vancomycin resistance. The inability to determine planktonic antibiotic susceptibility remains a limitation for molecular methods, and the inability to determine the sensitivity of biofilm bacteria is a limitation for both culture and molecular testing. However, the genetic component of antibiotic resistance is able to be determined using molecular testing [41].

Molecular diagnostics have proven useful at the Southwest Regional Wound Care Center in the past [22,23]. Published retrospective studies compared the outcomes of chronic wound patients using culture-based diagnostics in which, over a 7 month period 48% of the wounds healed. In contrast, using molecular bacterial diagnostics (Pathogenius Laboratory, Lubbock, TX) and therapies that target the detected microbes, more than 90% of wounds healed within a similar 7 month period. In another recent study to compare culture and molecular methods, it was shown that only comprehensive molecular methods could be correlated with clinical outcomes [26]. The weight of evidence is growing that suggests comprehensive molecular bacterial diagnostic methods can better serve chronic wound patients than culture testing.

## 5. Conclusions

Here we evaluated 168 wounds with cultures and molecular diagnostics. Out of the 168 wounds a total of 17 different bacteria were cultured. Molecular methods identified 338 different bacterial taxa. The majority of these were detected in relatively low abundance (~1% of the wound microbiota). Culture identified up to 3 different bacteria in some wounds, and molecular testing identified individual wounds with up to 33 different bacteria. The majority of bacteria identified with culture were also identified with molecular testing, but the vast majority of bacteria identified with molecular methods were not identified with culture methods. Although not tested for by culture, anaerobes were commonly found to be a large component of the microbial population in these chronic wounds, which is consistent with previous studies. In several instances, the culture methods identified the predominant populations as determined by molecular testing, but in the majority of the samples, culture underreported the diversity of the wound microbiota and failed to detect the most abundant bacteria in the wound. Previous studies have demonstrated that using the molecular identification of bacteria to guide the management of chronic wounds can improve patient outcomes.

## Figures and Tables

**Table 1:** An overview of wound types and most results for each wound type are listed. The most common results are at the top. Only the top 5 most common results for each wound type is presented. The number of wounds (# wounds) in each category is provided. Anaerobes are indicated (^*^), and anaerobes were not attempted to be detected by culture.

Type of wound	# wounds	Culture overview	Molecular overview
Decubitus ulcer	24	*Enterococcus*	*Corynebacterium*
		*Staphylococcus*	Peptoniphilus^*^
		*Pseudomonas*	*Staphylococcus*
		*Serratia*	Anaerococcus^*^
		*Proteus*	Bacteroides^*^

Diabetic extremity ulcer	40	*Enterococcus*	Anaerococcus^*^
		*Pseudomonas*	Peptoniphilus^*^
		*Streptococcus*	*Corynebacterium*
		*Serratia*	Finegoldia^*^
		*Staphylococcus*	*Pseudomonas*

Surgical site	23	*Staphylococcus*	*Corynebacterium*
		*Serratia*	*Staphylococcus*
		*Enterococcus*	Bacteroides^*^
		**no growth**	*Prevotella*
		*Pseudomonas*	*Serratia*

Venous leg ulcers	49	*Staphylococcus*	*Corynebacterium*
		**no growth**	*Staphylococcus*
		*Serratia*	Bacteroides^*^
		*Streptococcus*	*Prevotella*
		*Pseudomonas*	Peptoniphilus^*^

Trauma/abscesses	32	**no growth**	*Staphylococcus*
		*Staphylococcus*	*Prevotella*
		*Enterococcus*	Bacteroides^*^
		*Serratia*	Peptoniphilus^*^
		*Pseudomonas*	*Corynebacterium*

**Table 2:** The 17 bacterial taxa identified in 168 chronic wound samples using aerobic culture testing are listed. The number of wounds which were culture positive for each taxa are listed in the second column. The number of wounds which were positive for each taxa using 16S rDNA testing are listed in the third column.

Bacterial taxa detected from 168 samples using aerobic culture testing	Number of positive samples using culture	Number of positive samples using 16S sequencing
*Staphylococcus aureus*	41	72
*Enterococcus* spp.	35	28
*Serratia marcescens*	35	39
*Pseudomonas*	24	27
*Staphylococcus* spp. (not *S. aureus*)	20	47
*Streptococcus agalactiae*	18	23
*Proteus mirabilis*	9	10
*Citrobacter freundii*	5	6
*Escherichia coli*	4	3
*Klebsiella pneumoniae*	3	8
*Enterobacter aerogenes*	2	3
*Enterobacter cloacae*	2	3
*Morganella morganii*	2	5
*Streptococcus* spp.	2	41
*Xanthomonas maltophilia*	2	3
*Acinetobacter baumannii*	1	5
*Providencia* spp.	1	2

**Table 3:** The 20 most frequently detected bacteria in chronic wounds using DNA testing.

Bacteria identified using comprehensive molecular diagnostics	# of chronic wounds
*Staphylococcus aureus*^1^	72
*Finegoldia* magna 2	54
*Corynebacterium* striatum	53
*Anaerococcus* vaginalis 2	39
*Bacteroides vulgatus*^2^	39
*Serratia marcescens*^1^	39
*Prevotella* sp.	34
*Peptoniphilus harei*^2^	33
*Peptoniphilus ivorii*^2^	30
*Pseudomonas* sp. 1	27
*Anaerococcus* sp. 2	26
*Streptococcus agalactiae*. 1	23
*Klebsiella* sp.	18
*Prevotella* sp.	17
*Enterococcus* sp. 1	15
*Peptoniphilus* sp. 2	15
*Corynebacterium tuberculostearicum*	14
*Peptostreptococcus* sp. 2	14
*Clostridium cellobioparum*^2^	12
*Staphylococcus capitis*	11

Legend:

1 indicates bacteria in the top 20 bacteria identified by DNA testing which were also in the list of bacteria identified using culture methods;

2 indicates anaerobic bacteria, which were not attempted to be cultured.

**Table 4:** Results for 10 representative samples are reported. In addition to bacterial culture and 16S sequencing, PCR testing for bacteria and yeast, 18S sequencing for yeast, and identification of yeast during bacterial culture are also included in this table. The PCR testing examined each sample for *Candida albicans*, Group D *Enterococcus*, *Klebsiella pneumoniae*, *Pseudomonas aeruginosa*, *Staphylococcus* aureus, *Streptococcus agalactiae*, and *Streptococcus pyogenes*. The 16S/18S sequencing reports the presence of the organisms with a corresponding percentage. The percentage indicates the amount which the organism contributed to the whole of the microbiota, which was detected in the sample.

Sample	Culture	PCR	16S & 18S	%	Comments
1	*Pseudomonas aeruginosa*	*Pseudomonas aeruginosa*	*Pseudomonas aeruginosa*	65	Good agreement between all 3 testing methods
			Finegoldia magna*	30	
			Group D *Enterococcus*	<1	
			*Serratia marcescens*	<1	

2	*Streptococcus* spp.	*Streptococcus pyogenes*	Prevotella*	49	Streptococci were detected by culture, but staphylococci were missed by culture. The molecular methods are in agreement, but the predominant bacterium in the sample was not attempted to be detected by culture or PCR.
		*Streptococcus agalactiae*	*Staphylococcus aureus*	21	
		*Staphylococcus aureus*	Unknown Bacterium	16	
			*Streptococcus agalactiae*	9	
			*Streptococcus pyogenes*	4	

3	No Growth	*Klebsiella pneumoniae*	*Haemophilus parainfluenza*	33	The predominant bacterium was missed by culture and not attempted to be detected by PCR. Yeast and bacteria were both detected by molecular methods.
		*Candida albicans*	*Candida albicans*	15	
			*Klebsiella pneumoniae*	13	

4	*Pseudomonas* spp.	*Streptococcus pyogenes*	*Serratia* spp.	31	Culture and PCR detected two different organisms. 16S sequencing detected both organisms, but several other bacteria were present at a higher concentration in the sample than either of the bacteria detected by culture or PCR.
			Peptoniphilus harei*	28	
			*Corynebacterium striatum*	9	
			*Pseudomonas* spp.	5	
			*Streptococcus pyogenes*	<1	

5	*Serratia marcescens*	*Serratia marcescens*	*Corynebacterium striatum*	50	*Serratia* was detected by all methods, but 16S determined it to be a small contributor to the microbiota.
	*Proteus mirabilis*		Anaerococcus vaginalis*	19	
			*Serratia marcescens*	3	
			*Proteus* spp.	2	

6	*Staphylococcus aureus*	*Staphylococcu s aureus*	*Prevotella* spp. *	40	*Staphylococcus* was detected by all methods, but 16S determined it to be a very small contributor to the microbiota. The most predominant organisms were anaerobes that were not attempted to be detected by culture or PCR.
			Unknown Bacterium	32	
			Bacteroides vulgatus*	25	
			*Staphylococcus aureus*	<1	

7	*Acinetobacter baumannii*	Group D *Enterococcus*	*Staphylococcus* spp.	83	Culture and PCR detected different organisms, and 16S sequencing confirmed the presence of all of these organisms. PCR detected bacteria that were very small contribultors to the microbiota.
		*Streptococcus pyogenes*	*Acinetobacter baumannii*	6	
			*Streptococcus pyogenes*	<1	
			Group D *Enterococcus*	<1	

8	No Growth	Negative	Unknown Bacterium	91	The predominant bacterium is from an unknown bacterium

9	*Klebsiella pneumoniae*	*Klebsiella pneumoniae*	*Klebsiella pneumoniae*	43	All methods agree that *Klebsiella* and *Enterococcus* are present in the sample. PCR detected *Staphylococcus*, which 16S sequencing determined to be a very small contributor to the microbiota.
	*Enterococcus* spp.	Group D *Enterococcus*	Unknown Bacterium	23	
		*Staphylococcus aureus*	Group D *Enterococcus*	16	
			*Veillonella* spp. *	16	
			*Staphylococcus aureus*	<1	

10	Yeast	*Candida albicans*	*Candida albicans*	63	All methods detected yeast. Molecular methods also detected *Staphylococcus*.
		*Staphylococcus aureus*	*Staphylococcus aureus*	12	

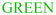
: Organism detected with all three testing methods;

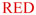
: Organism detected with two only two testing methods;

*: Bacterium is an obligate anaerobe.

No obligate anaerobes were attempted to be detected by culture or PCR.
